# Association of serum complement C3 level with disease severity in primary pterygium

**DOI:** 10.3389/fmed.2025.1671768

**Published:** 2025-09-23

**Authors:** Young In Yun, Jung Sun Heo, Seung Hyeun Lee, Kyoung Woo Kim

**Affiliations:** ^1^Department of Ophthalmology, Dongguk University Ilsan Hospital, Goyang, Republic of Korea; ^2^Department of Ophthalmology, Chung-Ang University College of Medicine, Chung-Ang University Hospital, Seoul, Republic of Korea; ^3^Department of Ophthalmology, Chung-Ang University College of Medicine, Chung-Ang University Gwangmyeong Hospital, Gwangmyeong, Republic of Korea

**Keywords:** complement C3, disease severity, serum biomarkers, pterygium, T grade

## Abstract

**Purpose:**

To investigate whether various systemic inflammatory and immunologic markers—including complement C3, C4, antinuclear antibodies (ANA), rheumatoid factor (RF), and other autoantibodies—are associated with the clinical severity of primary nasal pterygium.

**Methods:**

We retrospectively reviewed 26 eyes from patients with primary nasal pterygium. Serum complement levels (C3, C4), erythrocyte sedimentation rate (ESR), and autoimmune markers (ANA, RF, perinuclear anti-neutrophil cytoplasmic antibody [P-ANCA], cytoplasmic ANCA [C-ANCA], human leukocyte antigen [HLA]-B51, HLA-B27, anti-Ro [SSA], and anti-La [SSB]) were measured. Pterygium severity was graded using T (stromal translucency), V (vascularity), and the loss of plica semilunaris (LPS).

**Results:**

Among 26 patients (mean age 52.9 ± 14.9 years; 42.3% female), 61.5% tested positive for ANA. However, neither ANA positivity nor titer correlated with T, V, or LPS grades. Five patients (19.2%) had low C3 (<90 mg/dL). Although C4 and ESR did not correlate with disease severity, C3 levels showed a significant inverse correlation with the T grade (*r* = −0.477, *p* = 0.014). No significant association was found between C3 and the V grade or LPS, suggesting that severe stromal changes (T3) may be linked to modest complement consumption.

**Conclusion:**

Lower serum C3 levels were associated with advanced stromal opacification in pterygium, indicating possible complement activation in severe disease. While ANA was frequently positive, it did not correlate with clinical severity. These findings suggest that complement C3 may serve as a potential biomarker for advanced pterygium.

## Introduction

Pterygium is a relatively common ocular surface lesion involving fibrovascular tissue that extends from the conjunctiva onto the cornea, frequently attributed to chronic ultraviolet (UV) exposure and localized degenerative changes at the limbal region (1, 2). However, mounting evidence indicates that the pathogenesis of pterygium extends beyond simple UV-induced damage, encompassing chronic inflammation, immune reaction, extracellular matrix remodeling, and, in some cases, possible oncogenic or genetic predispositions (3–9). Several studies have detected elevated levels of inflammatory mediators such as interleukins, growth factors, and matrix metalloproteinases in pterygium tissue, underscoring the condition’s inflammatory component (2, 5, 10, 11).

In particular, recent investigations suggest that localized inflammatory cells and cytokines drive fibrovascular changes, and systemic immunologic factors may further amplify or perpetuate these pathological processes, influencing both the severity and recurrence of pterygium after surgical intervention (12–14). Such systemic factors could include low-grade autoimmune tendencies or exaggerated repair mechanisms triggered by continuous epithelial damage, suggesting that the interplay between ocular surface signals and systemic immune responses might be more critical than previously thought (15, 16). Laboratory markers such as serum complement components (C3, C4), rheumatoid factor (RF), and antinuclear antibodies (ANA) can reveal subtle systemic inflammation or autoimmune tendencies (17, 18). In particular, the complement system functions at the intersection of innate and adaptive immunity, potentially linking local tissue injury to broader systemic responses (19). Clinically, pterygium severity is often evaluated using the T grade (translucency), V grade (vascularity), and the loss of plica semilunaris (LPS) (6, 7, 20, 21). However, it remains unclear which, if any, of these severity scales correspond to systemic immune changes.

In the present study, we assessed serum complements including C3, C4, ESR and other common laboratory markers of autoimmune-related antibodies such as ANA, RF, human leukocyte antigen (HLA)-B27, HLA-B51, anti-neutrophil cytoplasmic antibody (ANCA), anti-Ro (SSA), and anti-La (SSB) antibodies in patients with primary nasal pterygium who had no known autoimmune disease. We aimed to determine whether any of these markers correlated with pterygium severity, as quantified by T, V, and LPS. In particular, we hypothesized that if local fibrovascular proliferation in advanced pterygium (e.g., T3) reflects robust inflammatory activity, then mild complement consumption might be observed.

## Materials and methods

### Study design and ethical approval

This retrospective cohort study was conducted at a single tertiary referral center and was based on a review of electronic medical records. The study protocol adhered to the ethical principles outlined in the Declaration of Helsinki. Approval for the study was obtained from the Institutional Review Board (IRB) of Chung-Ang University Hospital (Approval No. 2209–003-19434). Due to the retrospective nature of the investigation, the requirement for informed consent was waived by the IRB.

### Subjects

Asian adults diagnosed with primary nasal pterygium on slit-lamp examination who underwent blood testing to screen for autoimmune conditions were included in this study. Blood testing for autoimmune status was not performed due to clinical suspicion of a systemic autoimmune or inflammatory disease; rather, additional autoimmune laboratory assessments were optionally conducted at the patient’s request, within 1 month prior to scheduled pterygium surgery, as part of routine preoperative evaluation. Individuals were excluded if they presented with recurrent or temporal pterygia, had a known autoimmune disease (e.g., rheumatoid arthritis, systemic lupus erythematosus), used topical or systemic immunosuppressive medications (including corticosteroids) within the previous 3 months, or had other active systemic inflammatory disorders.

### Laboratory testing

Peripheral blood samples were obtained preoperatively to assess key inflammatory and immunologic markers. All laboratory tests were performed by the Department of Laboratory Medicine at our institution. To minimize potential bias in the test results, patients were assessed at the time of blood sampling for any acute conditions, including upper respiratory infections, menstruation, gastroenteritis, or other systemic inflammatory diseases. Subjects found to have any of these conditions were excluded from blood sample data collection for this study.

The following parameters were collected: ANA positivity and titer, RF concentration (IU/mL), perinuclear anti-neutrophil cytoplasmic antibody (P-ANCA; MPO Ab) and cytoplasmic ANCA (C-ANCA; PR3 Ab) positivity, HLA-B51 and HLA-B27 positivity, anti-Ro (SSA) and anti-La (SSB) antibody positivity, serum complement C3 and C4 concentrations (mg/dL), and ESR value (mm/h). Specifically, ANA testing was performed using the standard HEp-2 cell indirect immunofluorescence assay, and titers were reported at dilutions of 1:80 and 1:160 (22).

Reference ranges used were based on institutional laboratory standards: ESR, 0–20 mm/h; RF, <15 IU/mL; complement C3, 90–180 mg/dL; complement C4, 10–40 mg/dL. P-ANCA and C-ANCA titers were interpreted as negative (≤0.90), equivocal (0.91–1.09), or positive (≥1.10). Anti-Ro (SSA) and anti-La (SSB) antibody titers were defined as negative (<7 U/mL), equivocal (7–10 U/mL), or positive (>10 U/mL). All data were securely recorded in a dedicated database along with the clinical and demographic information of each subject.

### Clinical severity grading of pterygium

To evaluate the clinical severity of pterygium, all affected eyes were assessed using three established grading systems that address different morphological characteristics. These included: (1) the degree of stromal translucency of the pterygium body, known as the T grading system (21); (2) the extent of vascularization, classified by the V grading system (7); and (3) the vertical elongation of the plica semilunaris, referred to as the LPS grading system (20). Each grading method was applied with the aid of standardized reference photographs, ensuring consistency in evaluation. These grading systems are regarded as reliable tools for pterygium classification due to their use of objective visual standards or reproducible estimation techniques (23). To maintain consistency, all clinical grading was performed by a single examiner.

The T grading system stratifies pterygia into three categories—T1, T2, and T3—based on the visibility of underlying episcleral vessels, reflecting increasing stromal opacity. Likewise, the V grading system categorizes vascularity into V1, V2, and V3, corresponding to minimal, moderate, and severe vascular proliferation. For LPS analysis, slit-lamp images at ×10 magnification were captured during lateral gaze, both with and without a yellow barrier filter. The LPS extent was quantified by calculating the proportion of vertical elongation relative to the full length of the plica semilunaris, expressed as a percentage, based on previously described methodology (20).

### Outcome measurements and data analysis

Data analysis was performed based on the following scheme:

1. The positivity of serum autoantibodies and concentrations of RF, C3, C4, and ESR.
2. The association of ANA positivity with the clinical severity of pterygium (T, V grades and LPS).
3. The correlation of the serum concentration of C3, C4 and blood ESR levels with the clinical severity of pterygium.

### Statistical analysis

Prism software (GraphPad, La Jolla, CA, United States) was used for statistical tests. Continuous variables were expressed as mean ± standard deviation. To assess differences in continuous variables across the three groups, analysis of variance (ANOVA) was applied. Categorical variables were compared using the chi-square test. The correlation test was performed using parametric Pearson’s correlation test or non-parametric Spearman’s rank correlation test. A *p* value <0.05 was considered statistically significant.

## Results

### Demographics and clinical severities of primary pterygium

A total of 26 eyes from 26 patients satisfied the inclusion criteria. The average age was 52.9 ± 14.9 years, and 42.3% were female. Most participants presented with either T1 or T2 pterygia, although T3 lesions were also noted in a minority. Vascularity grading (V1, V2, V3) was similarly varied, with proportions roughly distributed across mild, moderate, and marked vessel engorgement. The mean LPS ratio was approximately 49.4 ± 35.3%, indicating moderate morphological distortion in many patients (Table 1). Notably, no patient had a documented history of autoimmune disease or prior immunosuppressant use.

**Table 1:** Demographics and baseline clinical severity indices of pterygium in subjects with primary nasal pterygium enrolled in this study.

Variables	Value
No. of subjects	26 eyes from 26 subjects
Age (yrs)	52.9 ± 14.9
% Female (M: F)	42.3% (15:11)
Clinical severity indices of pterygium	
T grade	
T1	9 eyes (34.6%)
T2	13 eyes (50.0%)
T3	4 eyes (15.4%)
V grade	
V1	3 eyes (11.5%)
V2	18 eyes (69.2%)
V3	5 eyes (19.2%)
Extent of LPS (%)	49.4 ± 35.3

Data shows mean ± standard deviation. LPS, loss of vertical length of plica semilunaris.

### Laboratory findings: autoimmune markers, complement, and ESR

A total of 61.5% of subjects tested positive for ANA (Table 2), notably exceeding previously reported epidemiologic rates of approximately 25% in healthy adult populations (24, 25). As detailed in Table 2, ANA titers varied from mild to moderate positivity. Conversely, other autoimmune-related markers— including RF, HLA-B27, HLA-B51, P- and C-ANCA, anti-SSA/Ro, and anti-SSB/La— exhibited low positivity rates, aligning with established background frequencies in asymptomatic adults. RF was positive in only a small proportion of participants, while anti-SSA/Ro and anti-SSB/La were rarely detected, suggesting limited additional autoimmune serologic abnormalities beyond ANA.

**Table 2:** Positivity of various serum autoantibodies in subjects with primary nasal pterygium enrolled in this study.

Serum antibodies	No. of subjects		
	Negative	Positive	
ANA	10 (38.5%)	16 (61.5%)	
		Titer 1:80	Titer 1:160
		12 (46.2%)	4 (15.4%)
P-ANCA	26 (100%)	0 (0%)	
C-ANCA	25 (96.2%)	1 (3.8%)	
HLA-B27	24 (92.3%)	2 (7.7%)	
HLA-B51	22 (84.6%)	4 (15.4%)	
Anti-Ro (SSA) antibody	25 (96.2%)	1 (3.8%)	
Anti-La (SSB) antibody	26 (100%)	0 (0%)	
	Within normal range ( < 15 IU/mL)	Elevated ( > 15 IU/mL)	
RF	23 (88.5%)	3 (11.5%)	
(IU/mL)	-	36.0 ± 20.6	

ANA, antinuclear antibody; ANCA, anti-neutrophil cytoplasmic antibody; P-ANCA, perinuclear ANCA; C-ANCA, cytoplasmic ANCA; HLA, human leukocyte antigen; RF, rheumatoid factor. Data shows mean ± standard deviation.

Serum complement levels generally fell within normal ranges (Table 3). Notably, serum C3 had a mean within the standard reference interval, yet 5 of the 26 participants (19.2%) showed levels below 90 mg/dL, with an average of 80.1 ± 3.5 mg/dL, suggesting possible complement consumption or mild systemic inflammatory responses. In contrast, no subject had a low serum C4 level, showing no evidence of depletion. Additionally, the mean ESR, a general inflammatory marker, was within normal limits. Although these findings point to minimal systemic autoimmune or inflammatory activation in most individuals, the subgroup displaying low C3 may point to subtle complement involvement in a fraction of patients with primary nasal pterygium.

**Table 3:** Serum concentration of complement C3 and C4 and erythrocyte sedimentation rate (ESR) in subjects with primary nasal pterygium enrolled in this study.

Serum markers	Value
C3 (mg/dL)	104.2 ± 18.0
C4 (mg/dL)	25.3 ± 8.0
ESR (mm/h)	16.7 ± 11.1

Data shows mean ± standard deviation. ESR, erythrocyte sedimentation rate.

### ANA positivity and clinical severity of pterygium

Based on the observation that 61.5% of our cohort tested positive for ANA, a rate higher than in the general population (Table 2), we examined whether ANA positivity and, if positive, its titer were associated with the three pterygium severity grading systems (T, V, and LPS). Although ANA-positive patients—particularly those with an ANA titer of 1:160—tended to show an increased LPS (%), the difference was not statistically significant (*p* = 0.879, ANOVA). Moreover, there was no significant association between ANA positivity and T grade or V grade (*p* = 0.770 and *p* = 0.101, respectively, chi-square test; Table 4).

**Table 4:** Association of the positivity of antinuclear antibody (ANA) and their titers with clinical severity indices of pterygium in subjects with primary nasal pterygium enrolled in this study.

Variables	No. of subjects								LPS (%)^b^	*p* value
	T grade^a^				V grade^a^					
	T1	T2	T3	*p* value	V1	V2	V3	*p* value		
ANA										
Negative	4 (44.4%)	4 (30.8%)	2 (50%)	0.770	0 (0%)	8 (44.4%)	2 (40%)	0.101	46.5 ± 39.4	0.879
Positive (1:80)	3 (33.3%)	7 (53.8%)	2 (50%)		1 (33.3%)	8 (44.4%)	3 (60%)		49.2 ± 32.5	
Positive (1:160)	2 (22.2%)	2 (15.4%)	0 (0%)		2 (66.7%)	2 (11.1%)	0 (0%)		57.5 ± 41.9	
Total	9 (100%)	13 (100%)	4 (100%)		3 (100%)	18 (100%)	5 (100%)		−	−

Data shows mean ± standard deviation. ANA, antinuclear antibody; LPS, loss of vertical length of plica semilunaris.

a Chi-square test.

b Analysis of variance.

### Correlation of complements and ESR with clinical severity of pterygium

**Figure 1:**
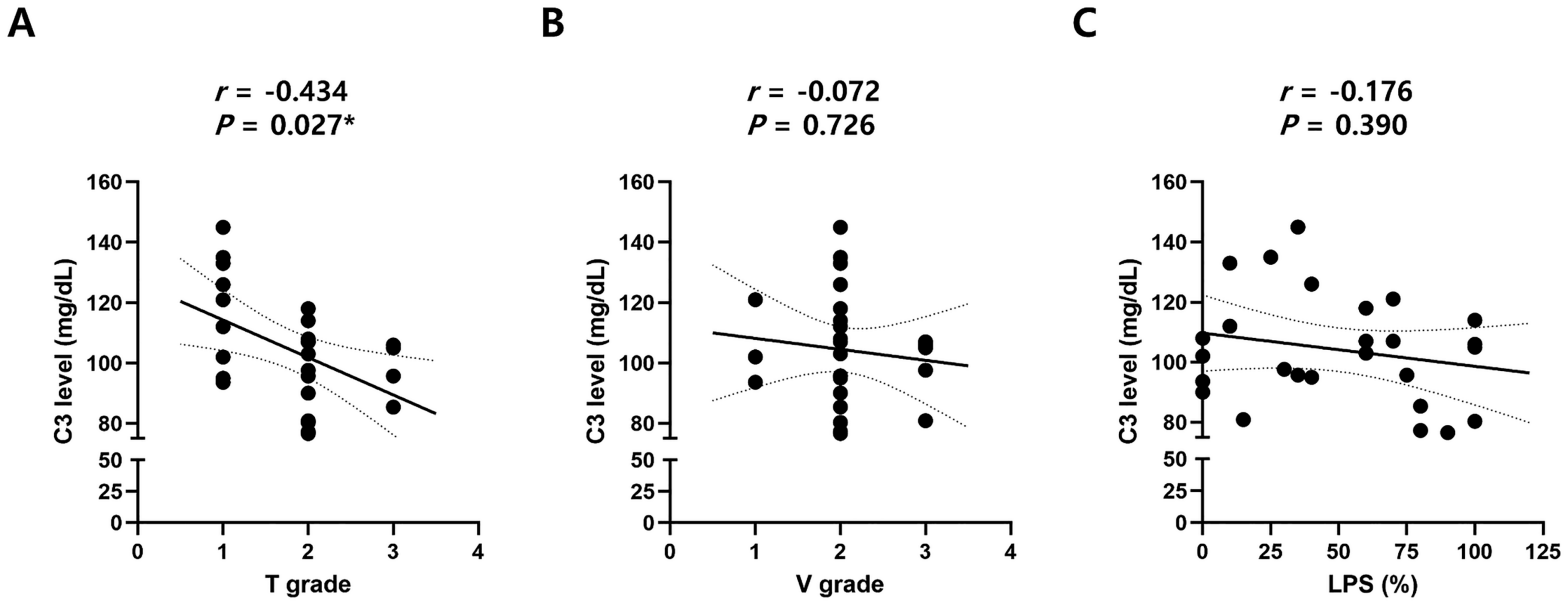
Correlation between serum complement component 3 (C3) levels and clinical severity indices of pterygium. Scatter plots with regression lines showing correlation between serum C3 and **(A)** the degree of stromal translucency of the pterygium body (T grade), **(B)** the extent of vascularization (V grade), and **(C)** the vertical elongation of the plica semilunaris (LPS). *r*, correlation coefficient; *p*, *p*-value; *p-value < 0.05. Statistically significant values (*p* < 0.05) are shown in asterisk (*).

Whereas complement C4 and ESR showed no correlation with T grade, V grade, or LPS, complement C3 demonstrated a significant negative correlation with T grade (*r* = −0.434, *p* = 0.027, Spearman’s rank correlation test; Table 5; Figure 1). In contrast, neither the V grade nor the LPS exhibited a significant association with serum C3 levels. This distinctive finding in T3 eyes suggests that severe stromal overgrowth may be accompanied by modest complement activation or consumption at the systemic level.

**Table 5:** Correlation between serum markers including complements C3 and C4, and erythrocyte sedimentation rate (ESR) concentrations with clinical severity indices of pterygium in subjects with primary nasal pterygium enrolled in this study.

Serum markers vs.		T grade	V grade	LPS (%)
C3	*r*	−0.434	−0.072	−0.176
	*p* value	0.027*	0.726	0.390
C4	*r*	0.087	0.301	−0.142
	*p* value	0.672	0.136	0.489
ESR	*r*	−0.042	−0.145	−0.142
	*p* value	0.838	0.480	0.489

LPS, loss of vertical length of plica semilunaris. ESR, erythrocyte sedimentation rate. r, Spearman’s rank correlation coefficient. *p < 0.05 by Spearman’s rank correlation test.

## Discussion

In this retrospective cohort study, we investigated whether systemic inflammatory and immunologic markers might correlate with the severity of primary nasal pterygium, as evaluated by three established grading systems (T, V, and LPS). Our main finding was a notable inverse relationship between serum complement C3 levels and the T grading of pterygium, suggesting that more pronounced stromal opacification (T3) may reflect greater complement consumption or activation. In contrast, neither complement C4, ESR, nor the presence of ANA correlated significantly with the severity of pterygium, based on T, V, or LPS metrics.

From a pathophysiological standpoint, pterygium is widely regarded as a localized fibrovascular overgrowth of conjunctival tissue onto the cornea, often attributed to chronic UV exposure and degenerative processes (1, 26). However, recent evidence has highlighted a more complex pathogenesis involving chronic inflammation, immune-cell infiltration, altered growth factor signaling, and extracellular matrix remodeling (27–29). Notably, our results align with emerging data suggesting that the immune response in pterygium may not be confined solely to the ocular surface. Rather, it may extend systemically via signaling pathways that involve cytokines, chemokines, and complement factors as partially revealed previously (12, 30). The significant negative correlation between serum C3 and T grade in our cohort provides a novel perspective, implying that local inflammatory events in advanced pterygium can manifest as measurable systemic alterations in complement components.

The complement system, part of the innate immune defense, plays a pivotal role in bridging innate and adaptive immunity (31). In ocular conditions such as age-related macular degeneration and certain forms of uveitis, dysregulation of complement pathways has been implicated in disease progression (32, 33). In the context of pterygium, complement activation may be triggered by ongoing tissue injury, immune complex deposition, or the release of inflammatory mediators from conjunctival fibroblasts and infiltrating leukocytes. In our study, reductions in C3 without parallel decreases in C4 suggest a pathway-specific pattern of activation. Because C3 is the central convergence point of all three complement pathways (classical, lectin, and alternative), whereas C4 reflects only classical and lectin activity, this finding points toward alternative pathway activation or nonspecific complement amplification rather than classical pathway activity alone—a pattern also observed in other alternative pathway–driven ocular disease such as age-related macular degeneration (31, 34–36). Although our data do not pinpoint the exact mechanisms, the selective reduction in C3 highlights a potentially key immunologic process underlying severe pterygium. To our knowledge, no prior studies have demonstrated a link between a systemic complement component and clinical severity indices in pterygium. This highlights the novel contribution of our study and suggests that serum C3 may serve as a tentative biomarker for identifying patients with more aggressive, “fleshy” pterygia, with potential relevance for prognosis and follow-up.

Despite the observed alterations in C3, other systemic autoimmune markers in our cohort such as ANA and RF did not show a clear relationship with pterygium severity. Interestingly, more than half (61.5%) of our subjects tested positive for ANA, a rate higher than that reported in the general population, which is typically around 25% (24, 25). Several factors may account for this discrepancy: ANA prevalence is known to increase with age, and since pterygium predominantly affects older adults—58% of our cohort were over 50 years and 35% over 60—age-related effects may partly explain the elevated rate (37–39). ANA prevalence also varies across ethnic and geographic backgrounds; for example, Korea has a relatively high prevalence of tuberculosis and latent tuberculosis infection compared to Western countries, and such chronic infections may contribute to immune activation (40–42). In addition, ANA rates differ depending on assay methodology and cut-off thresholds, which vary between laboratories. For instance, some laboratories consider titers of ≥1:160 as clinically significant, whereas we defined ANA positivity as ≥1:80, which may have inflated the overall positivity rate; however, the proportion of patients with ANA ≥1:160 in our cohort was substantially lower (24, 43).

The clinical significance of this elevated ANA positivity remains uncertain, particularly given the lack of correlation with T, V, or LPS grades. Another possible explanation is that ANA positivity in these patients might reflect a subclinical or low-grade immune dysregulation rather than an overt systemic autoimmune disease. ANA can be present in healthy individuals, and may fluctuate with transient inflammatory states (44). Moreover, low-titer ANA does not necessarily confer a diagnosis of autoimmune disease, and its pathogenic relevance can vary considerably (45). Thus, while our findings underscore a possible systemic immune component in some patients with primary pterygium, ANA positivity alone cannot be taken as evidence of clinically significant autoimmune activity in this setting.

When interpreting our results, it is also essential to consider the strengths and limitations of the pterygium grading systems employed. The T, V, and LPS scoring methods each capture distinct morphological or vascular characteristics. Specifically, the T grading system focuses on the translucency of the pterygium body and the visibility of underlying episcleral vessels, potentially providing a direct measure of stromal opacification that correlates with inflammatory and fibrovascular activity (21). Meanwhile, V grade emphasizes vascular proliferation, and the LPS index measures vertical distortion of the plica semilunaris (7, 20). The selective correlation of C3 with T grade, but not with V grade or LPS, suggests that stromal changes in pterygium may be especially relevant to immune activation at the systemic level. This discrepancy raises questions about whether vascularity and plica elongation are primarily driven by local factors (e.g., angiogenic stimuli) rather than broader immune complex formation or complement-mediated pathways.

From a clinical perspective, our findings propose that serum C3 may serve as a potential biomarker, albeit not a definitive one, to identify patients with more aggressive pterygium. Such information could aid in tailored treatment approaches or close postoperative monitoring, given that severe stromal changes often predict a higher risk of recurrence after excision (6, 21). Nevertheless, additional large-scale prospective studies are warranted to validate the use of C3 levels as a prognostic tool. Expanding the evaluation to include complement regulatory proteins (e.g., factor H, factor B), cytokine panels, and local tear-fluid analyses might further clarify the mechanistic links between pterygium severity and systemic immunologic alterations.

This study is not without limitations. First, our cohort was relatively small, which may limit the generalizability of the findings and preclude more definitive conclusions regarding the prognostic utility of C3. Second, the local ocular complement activity, such as in tears or aqueous humor, was not assessed in this study; future studies incorporating local complement activity analysis would provide more mechanistic insight. Third, only a subset of individuals underwent comprehensive autoimmune workups, and these tests were performed primarily at the patient’s request, leading to a potential selection bias. Prospective research designs with standardized immunologic testing across larger, more diverse populations would provide a more robust evaluation of complement pathways in pterygium pathogenesis.

In conclusion, our investigation suggests that higher T grade pterygium may be linked to a modest decrease in serum C3 levels, pointing to a potential role of systemic complement activation in the pathophysiology of advanced pterygium. While serum ANA positivity was relatively common, its clinical significance in relation to pterygium severity remains unclear. Future studies aimed at delineating the complex interplay between local ocular surface changes and systemic immunologic factors could pave the way for improved risk stratification and therapeutic interventions.

## Data Availability

The raw data supporting the conclusions of this article will be made available by the authors, without undue reservation.
